# Decreased inter-hemispheric interactions but increased intra-hemispheric integration during typical aging

**DOI:** 10.18632/aging.102421

**Published:** 2019-11-21

**Authors:** Qunlin Chen, Yunman Xia, Kaixiang Zhuang, Xinran Wu, Guangyuan Liu, Jiang Qiu

**Affiliations:** 1 School of Mathematics and Statistics, Southwest University, Chongqing, China; 2 Key Laboratory of Cognition and Personality, Ministry of Education, Chongqing, China; 3 School of Psychology, Southwest University, Chongqing, China; 4 State Key Laboratory of Cognitive Neuroscience and Learning, Beijing Normal University, Beijing, China; 5 College of Electronic and Information Engineering, Southwest University, Chongqing, China

**Keywords:** aging, resting-state fMRI, functional connectivity, hemispheric interaction

## Abstract

Normal aging is known to be accompanied by decreased segregation across the whole-brain functional network, which is associated with cognitive decline. Although compelling evidence supports reduced segregation and increased integration in whole-brain functional connectivity with aging, the age effect on the reorganization of large-scale functional networks at the hemispheric level remains unclear. Here, we aimed to examine age-related differences in inter-hemispheric interactions and intra-hemispheric integration by using resting-state functional MRI data of a healthy adult lifespan sample. The results showed that age-related decreases in inter-hemispheric integration were found in entire functional networks in both hemispheres, except for the sensorimotor network (SMN) and posterior default mode network (DMN). Specifically, aging was accompanied by increasing inter-hemispheric segregation in the left frontoparietal network (FPN) and left ventral attention network (VAN), as well as right-brain networks located in the auditory network (AN), visual network (VN), and temporal parts of the DMN. Moreover, aging was associated with increasing intra-hemispheric integration within the bilateral VN and posterior DMN while decreasing intra-hemispheric integration within the right VAN. These remarkable changes with aging confirm that there are dynamic interactions between functional networks across the lifespan and provide a means of investigating the mechanisms of cognitive aging.

## INTRODUCTION

Population aging is unexpectedly increasing throughout the world, and it has also been a major focus of research in the neuroimaging field [1]. Over the past decades, a large body of research has indicated that typical aging is characterized by localized degeneration in brain structure and widespread changes in functional brain activity, which have been associated with cognitive decline, such as attention, executive function, and memory [2–6]. Increasing evidence has suggested that age-related cognitive decline was related to the multiscale network of complex activity patterns instead of an ensemble of an isolated brain region [7–9]. Meanwhile, with the development of neuroimaging techniques, resting-state functional magnetic resonance imaging (rs-fMRI) has become an important approach to investigate intrinsic brain activity in the human brain because of its simplicity (e.g., no particular task) and the reliability of fMRI data acquired at rest [1, 10, 11]. Thus, rs-fMRI is a promising way to investigate brain functional network changes in typical aging.

A common finding among rs-fMRI studies has indicated that aging is associated with decreased resting-state functional connectivity (rsFC) in some brain networks [1, 9, 12] and the topological properties of the entire brain network [13]. In general, older adults showed decreased modularity and local efficiency compared to young and middle-aged adults [2, 4, 12, 13]. Remarkably, these brain networks (e.g., default mode network and executive control network) supporting high-order cognitive functions showed a considerably decreased connectivity with normal aging. For example, most studies have documented severely disrupted functional connectivity in the default mode network in older adults compared with younger adults and has also been considered as a neural marker in Alzheimer's disease [14–16]. Although it has been commonly reported that rsFC declines with aging, several studies have shown that the primary systems (e.g., sensorimotor network and visual network) that are responsible for the processing of sensory input and motor output show increased functional connectivity with aging [13, 17]. In addition, some studies have found that increased inter-network connectivity, especially in prefrontal regions, was associated with better cognitive performance in older adults [18], indicating that functional connectivity changes with aging are not always straightforward.

Regarding the decreased and increased functional connectivity in aging, several theories aim to provide an integrative interpretation for brain function and cognition changes in aging, in which one classical view—the hemispheric asymmetry reduction in old adults (HAROLD) model—has pointed out a compensatory function between hemispheres in older adults in that a less lateralized pattern of activity can counteract age-related neurocognitive decline [19]. In fact, hemispheric lateralization or asymmetry between the two hemispheres is viewed as an evolutionarily conserved mechanism in the human brain that is implemented in the dominant processing of specific cognitive tasks, allowing fast and efficient information processing [20–22]. In aging research, symmetrical activation and homotopic rsFC changes have been typically interpreted as compensation [23]. One remarkable study using rs-fMRI data found that global homotopic rsFC increased in older adults [24], which to some extent supports the compensation hypotheses that aging is associated with decreased hemispheric asymmetry resulting in bilateral cooperation or less inhibition from homotopic regions. However, further exploration of intra- and inter-hemispheric functional connectivity changes across healthy aging remains lacking. It is necessary to investigate the functional connectivity changes with aging at the hemispheric level to more deeply understand the neural basis of aging and neurodegenerative diseases.

In sum, typical aging will weaken the rsFC in particular functional networks (i.e., attenuation segregation) and enhance the interaction between other functional networks (i.e., increased integration). However, it remains unclear whether intra- and inter-hemispheric segregation and integration occur across the healthy adult lifespan and whether there are distinguishing patterns between two hemispheres. Accordingly, in this cross-sectional study, we aimed to examine age-related differences in the interactions of inter- and intra-hemispheric resting-state functional networks based on the symmetrical cortical template of the human brain.

## RESULTS

**Figure 1:**
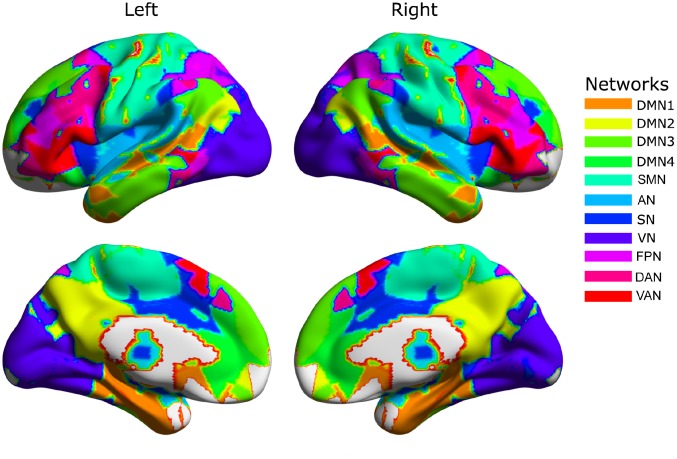
**The group-level community structure.** The default mode network (DMN) is divided into four major subdivisions: superior temporal cortex and medial temporal cortex (DMN1); precuneus, posterior cingulate cortex and lateral parietal cortex (DMN2); dorsal medial prefrontal cortex (DMN3); and ventral medial prefrontal cortex and lateral temporal cortex (DMN4). The remaining networks include the sensorimotor network (SMN), auditory network (AN), salience network (SN), visual network (VN), dorsal attention network (DAN), ventral attention network (VAN) and fronto-parietal network (FPN).

The group-level network partition across two hemispheres detected by the Louvain algorithm resulted in 11 functional networks (Figure 1) corresponding to the sensorimotor network (SMN), auditory network (AN), salience network (SN), visual network (VN), fronto-parietal network (FPN), dorsal attention network (DAN), ventral attention network (VAN), and four sub-networks of the default mode network (DMN), including the medial temporal lobe (MTL) and parts of the superior temporal cortex (DMN1), the posterior cingulate cortex and supramarginal gyrus (DMN2), the medial prefrontal cortex (DMN3), and the inferior parietal lobule, parts of lateral temporal cortex and superior medial prefrontal cortex (DMN4).

**Figure 2:**
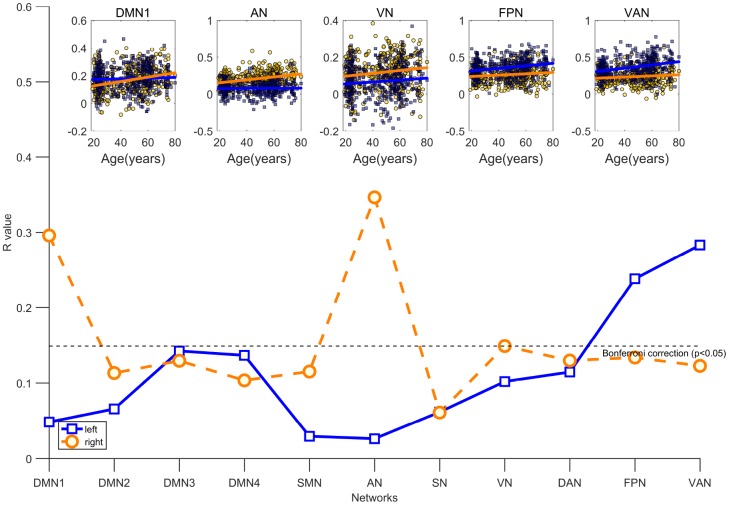
**Age is associated with increasing inter-hemispheric segregation of brain networks after controlling for the participant’s sex and mean FD.** The blue fitted line indicates that a given network in the left hemisphere is segregated from the right hemisphere; the orange fitted line indicates that a given network in the right hemisphere is segregated from the left hemisphere. All significant results survived Bonferroni correction at p <0.05.

To explore whether distinct patterns of age-related differences in inter-hemispheric segregation exist, we examined the linear relationships between hemispheric segregation of each community and age. As shown in Figure 2, this general pattern depicts that inter-hemispheric segregation increased with aging. Specifically, age was positively associated with inter-hemispheric segregation in the FPN (*r* = 0.24, P < 0.001) and VAN (*r* = 0.28, P < 0.001) in the left hemisphere, whereas age was positively associated with inter-hemispheric segregation of brain networks in the right hemisphere mainly located in the DMN1 (*r* = 0.30, P < 0.001), AN (*r* = 0.35, P < 0.001), and VN (*r* = 0.15, P = 0.002). After controlling for sex and mean FD, consistent relationships were found between age and inter-hemispheric segregation in these systems (Supplementary Table 1).

**Figure 3:**
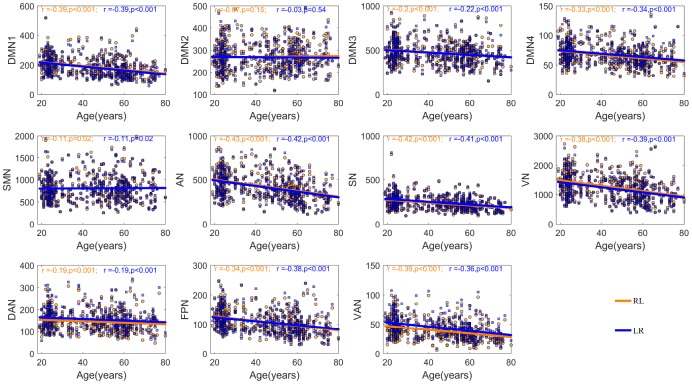
**The relationship between age and inter-hemispheric functional connectivity between homotopic networks after controlling for the participant’s sex and mean FD.** The blue fitted line indicates a given network in the left hemisphere interacted with the homotopic network in the right hemisphere; the orange fitted line indicates a given network in the right hemisphere interacted with the homotopic network in the left hemisphere.

Increasing inter-hemispheric segregation with age may reflect age-related decreases in inter-hemispheric functional communication. Thus, we further examined the linear relationships between inter-hemispheric integration of each community across the two hemispheres and aging. Figure 3 illustrates that aging is associated with the decreasing inter-hemispheric integration between most homotopic networks except the DMN2 and SMN, regardless of the direction of the connectivity. To investigate whether the age-related decreases in inter-hemispheric integration existed only between homotopic networks or also occurred between non-homotypic systems, we also explored the relationship between whole brain functional connectivity of a given network and age, and the results are consistent with preceding findings (Supplementary Figure 1).

**Figure 4:**
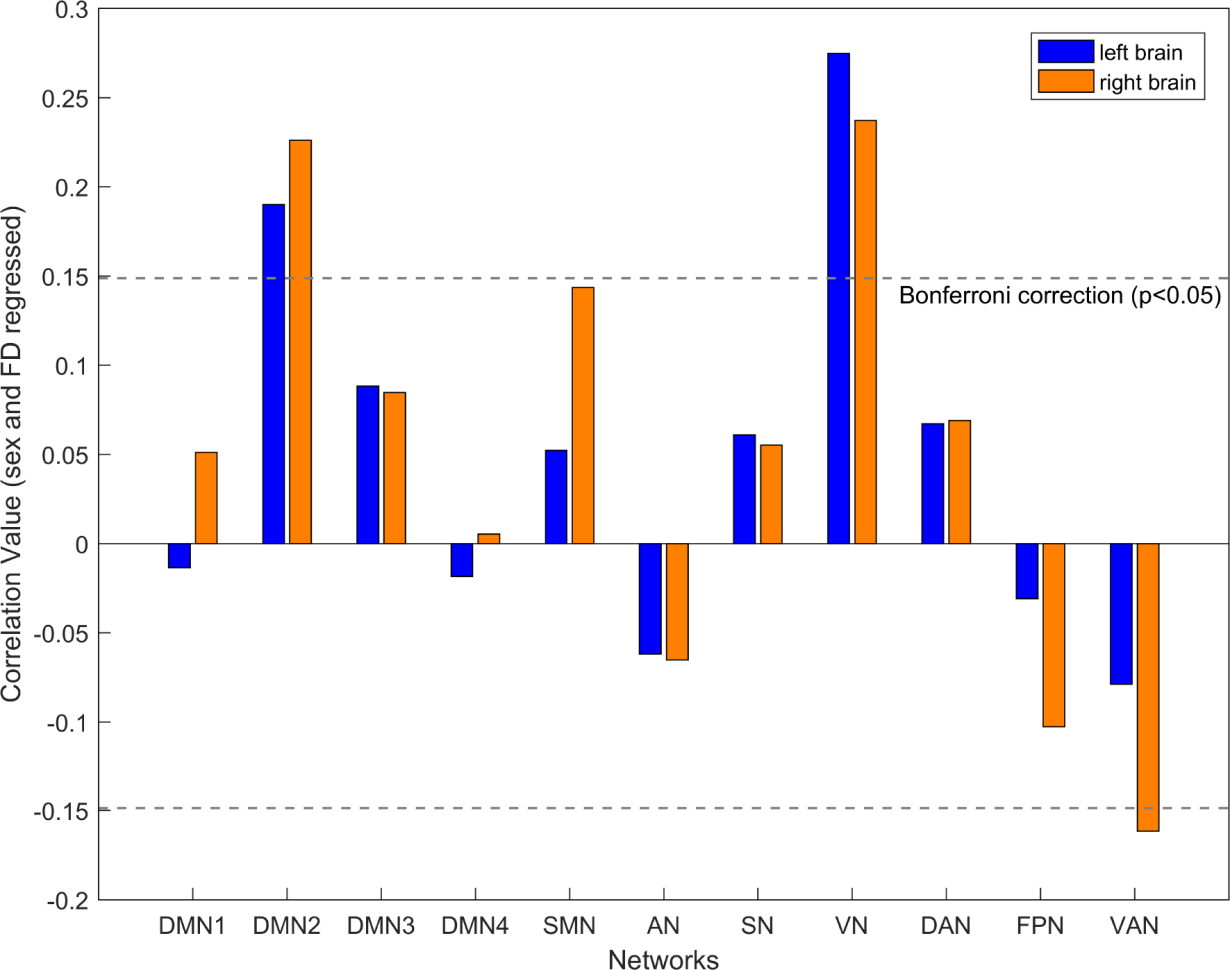
**The relationship between age and intra-hemispheric integration for each network after controlling for the participant’s sex and mean FD.** The blue box indicates the left hemisphere network, and the yellow box indicates the right hemisphere network. A significant correlation with the threshold of FDR-corrected p < 0.05.

Given the findings of age-related differences in inter-hemispheric interactions, it is inevitable to raise the question of whether aging also impacts the intra-hemispheric interaction. To this end, we examined intra-hemispheric integration changes with aging using the mean participation coefficient of the brain network within hemispheres. We assumed that functional integration within the hemisphere increased with age, as compensation for decreasing inter-hemispheric connectivity. As expected, age was positively associated with intra-hemispheric integration in the DMN2 (left: r = 0.19, P < 0.001; right: r = 0.23, P < 0.001) and VN (left: r = 0.27, P < 0.001; right: r = 0.24, P < 0.001) in both hemispheres. Additionally, age was negatively associated with intra-hemispheric integration in the right VAN (r = -0.16, P < 0.001) after controlling for sex and mean FD (Figure 4). Furthermore, validation analyses showed similar results: increasing intra-hemispheric integration in the bilateral DMN2 and VN, as well as decreasing intra-hemispheric integration in the right VAN with age across a range of edge densities (1-10%, see Supplementary Figure 2).

**Figure 5:**
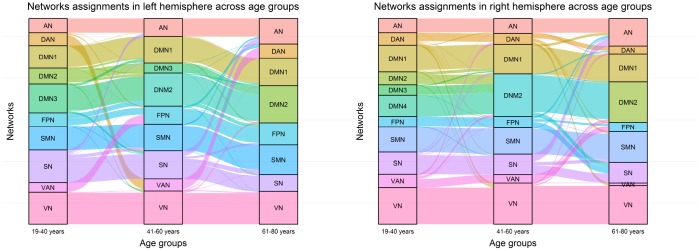
**Network assignment across three age groups.** The left alluvial diagram indicates community reorganization of the left brain regions with age; the right alluvial diagram indicates community reorganization of the right brain regions with age. Each block represents a network, and each line corresponds to a brain region.

To expand on the observations regarding the dynamics of intra-hemispheric integration with aging, we used an alluvial diagram to illustrate changes in community assignments of brain regions in each hemisphere across three age groups. As shown in Figure 5, the community number decreased with increasing age in both hemispheres. In early adults, the default system consisted of 3 sub-components in the left brain and 4 sub-components in the right brain, while in the elderly group, parts of the default system (e.g., DMN3 and DMN4) merged into the DMN2 and then generated a classical DMN, including the posterior cingulate cortex, supramarginal gyrus, medial prefrontal cortex, inferior parietal lobule, parts of the lateral temporal cortex, and superior medial prefrontal cortex. Notably, the community assignments of the DMN1 (including the medial temporal lobe and parts of the superior temporal cortex) remained stable across the three age groups. For the sensory-motor system, parts of regions within the SN were taken into the AN and SMN, while the VN remained highly stable across age groups. For the executive control system (including the FPN, DAN, and VAN), community assignments of brain regions dynamically changed across age groups only in the bilateral hemisphere, which was merged entirely into the FPN in the late adult.

## DISCUSSION

This study used cross-sectional resting-state fMRI data to characterize age-related effects in inter-and intra-hemispheric brain functional connectivity across the healthy adult lifespan. The present findings indicated that increased age is related to less inter-hemispheric functional connectivity between homotopic networks, which seemed to be a result of increased inter-hemispheric segregation with age in most networks. However, the age effect on inter-hemispheric segregation showed distinct patterns in the two hemispheres: the FPN and VAN in the left hemisphere and the AN, DMN1, and VN in the right hemisphere. In addition, increasing age was accompanied by increasing intra-hemispheric integration of brain networks located in the bilateral VN and posterior DMN, as well as decreasing intra-hemispheric integration in the right VAN. Further analysis showed that decreasing the number of communities with age in this process that the sub-components of the DMN gradually merged into a whole, parts of nodes in the SN were taken into the SMN, and the AN and VAN were integrated into the FPN. These findings provide hemisphere-level evidence of age-related functional degeneration of the brain across the adult lifespan. Below, we discuss the implications concerning these observations for understanding the functional reorganization of brain networks and cognition decline with aging.

First, most networks had less inter-hemisphere communication with aging except for the SMN and DMN2, which means aging is accompanied by the intensive attenuation of functional connectivity. Consistent findings have provided evidence that typically aging people present disrupted connectivity [23, 25]. It may usually be explained by the dedifferentiation hypothesis, which claims that the human brain will decrease functional connectivity and lose functional specificity in the aging process [26, 27]. This phenomenon may be a consequence of a decline in dopaminergic neuromodulation, which increases neural noise and reduces distinctive cortical representations [28]. However, the distinctive cortical representations may be implicated in cognitive abilities such as working memory and executive control abilities [28]. The decreased functional connectivity between the hemispheres may imply a disrupted whole-brain network and a reduced efficiency of information transfer between different brain regions. Regarding the SMN and DMN2, the unchanged interactions between the two hemispheres can be explained using a compensatory mechanism. The SMN is involved with motor tasks, and its altered functional connectivity has typically been associated with aging [29]. Previous studies have suggested that the segregation of the SMN is an age-related decrease, which is highly correlated with sensorimotor performance [29, 30]. Additionally, the DMN2, including the posterior cingulate cortex and supramarginal gyrus, is a region necessary for episodic memory and working memory that are vulnerable to aging [31–33]. The unchanged inter-hemispheric integration of SMN and DMN2 may be helpful to complete the sensorimotor and memory tasks during healthy aging. Collectively, there are large-scale decreases in the functional connectivity with typical aging; however, there is also a compensatory mechanism in the regions serving sensorimotor performance and episodic memory performance.

Although the communication between the hemispheres decreased in general, the networks with increasing functional segregation were not symmetrical in the two hemispheres. This study found that with aging, the FPN and VAN in the left hemisphere had reduced inter-hemispheric connectivity, while the DMN1, AN, and VN in the right hemisphere decreased functional connectivity with the opposite hemisphere. This asymmetric distribution of networks with increasing segregation may have been associated with lateralization-related changes in aging. The HAROLD claims that an age-related decrease in lateralization may result from the non-dominant hemisphere increasing activity as a compensatory mechanism. However, our findings seem to be inconsistent with an account based on the HAROLD model. Converging studies have proposed that the FPN is a right-hemisphere–lateralized network [34, 35]. In this study, the non-dominant hemisphere of the FPN did not show increased communication towards the opposite hemisphere as a compensatory effect; conversely, it decreases the connectivity between hemispheres. Therefore, we think that the increase in inter-hemispheric segregation in the left FPN cannot be explained by the HAROLD model. The decreased inter-hemispheric connectivity in the FPN may be a mechanism underlying executive control and working memory functional declines [36, 37]. In addition, the VAN is also right-hemisphere lateralized and associated with “top-down” attention control [38]. More specifically, the right VAN can control the attention shift to both sides, whereas the left VAN only controls the attention shift to the right side [39]. Corbetta et al. proposed that the output of the VAN may serve as a “circuit-breaker” for the automatic processing of the DAN, such that the attention is reoriented to the object that is significant for individuals [40]. Therefore, the increased inter-hemispheric segregation in the left VAN, i.e., decreased communication between the bilateral VAN, may imply decreased top-down attention control to the right side.

The DMN1 shows left lateralization and contains key regions for long-term memory and auditory processing [41]. The dominant hemisphere of the MTL is involved with verbal memory, and the non-dominant hemisphere is involved with non-verbal memory [42]. It was discovered that the bilateral superior temporal cortex was involved in processing when the environmental noise did not overwhelm the auditory object, although the individuals relied on the left hemisphere since background noise was too high [42].Therefore, the disruptions in the right DMN1 may reflect cognitive decline in non-verbal memory instead of verbal memory, as well as the ability to acquire target auditory information from a noisy background. Although HAROLD is the prevailing theory regarding decreased hemispheric asymmetry in aging, James et al. challenged this assumption by finding that lower asymmetry in aging was associated with less activation in the lateralized hemisphere instead of a compensatory effect of the contralateral hemisphere [43]. A previous study suggested that the AN is right-hemisphere lateralized and that the right AN is better integrated with the opposite hemisphere [44]. According to the proposal of James et al., we believe that the increased inter-hemispheric segregation of the right AN may imply a decrease in the integration ability of the dominant hemisphere of the AN. Similarly, there is evidence that the VN is strongly right-hemisphere lateralized and shows decreased right-hemisphere lateralization with age [45]. The increase in inter-hemispheric segregation of the right VN may indicate a disruption in the dominant hemisphere of the VN. These findings confirmed that there is a hemispheric difference in the impact of age on network interaction patterns. Distinguishing the aging effect on brain network interaction patterns between the two hemispheres is helpful for understanding the brain network foundation of cognitive aging.

On the other hand, some evidence has suggested that the whole-brain networks become more integrated with aging, which seems to contradict our findings [4]. These seemingly inconsistent findings may be because this study examined the age-related brain network interactions based on the hemispheric level instead of the whole-brain level. That is, the increased integration of whole-brain networks is the combination of the decreased integration of inter-hemispheric networks and increased integration of the intra-hemispheric networks. The further analysis of the results suggested that the intra-hemispheric integration in the DMN2 and VN increased with age, which also supports this hypothesis. Previous studies have shown that the FC between the anterior and posterior portions of the DMN might be a sensitive indicator of aging and are decreased in the elderly [46]. However, in this study, we divided the DMN into four sub-networks and calculated the intra-hemispheric integration. The connectivity between the DMN2 and other networks may have compensated for the decreased FC between the DMN2 and DMN4. The increased intra-hemispheric integration in the VN and DMN with ageing may be explained by a compensatory mechanism: the decreased inter-hemispheric communication led to a decreased efficiency in information transfer between different brain networks, thus the increased intra-hemispheric connectivity is required. In addition, in the aging process, the right VAN showed decreased intra-hemispheric integration, i.e., a disconnection between the right VAN and other networks, which correlated with the age-related decline in flexible attentional control [47].

Of additional interest, typical aging was accompanied by dynamic changes in community assignments within the two hemispheres. Concretely, the tendency for changes in the community assignments was similar between two hemispheres: the sub-components of the DMN gradually merged into a whole, parts of the nodes in the SN merged into the SMN and AN, and the VAN disappeared and blended into the FPN. First, we found that a decreased number of modules across age groups was in line with compelling evidence of age-related decreases in modularity due to decreased connections within specific functional networks or global changes throughout the networks [4, 13, 48]. Second, these results further support and provide more details about intra-hemispheric integration, especially the increasing intra-hemispheric integration in the DMN2, which means that other special functional regions within the default network (i.e., DMN3 and DMN4) were integrated with aging. Most aging-related fMRI research has focused on the DMN given that some disability-related regions are contained within it, such as the posterior cingulate cortex and the hippocampus, which have been viewed as critical neuropathological origins of Alzheimer’s disease [49, 50]. Consistent with this, the two networks—DMN1 (involved in the hippocampus) and DMN2 (including the posterior cingulate cortex)—maintained their own functional modularity with age but simultaneously needed to integrate other sub-components to maintain specific functions, such as memory performance [51]. Regarding functional modularity in the AN and SMN compensating by integrating the SN with age, one possible reason is that adjacent regions may be easily assimilated because of the spatial distance, which is consistent with increasing short-range functional connectivity density hubs in the somatosensory network with age [17]. Similar functional dedifferentiation in the executive control system, for example, the VAN involvement in detecting unattended or unexpected stimuli, was integrated into the FPN and implicated in top-down attention control and guided allocation of attention [52]. It is noteworthy that the modularity in the VN remained stable with age, which may represent high-level functional segregation with a specialized role in the human brain. Overall, these preserved networks have been critically implicated in primary information processing, executive functions, and memory, which supports basic cognitive requirements for daily life in older people.

Although our study extended the knowledge of brain functional connectivity in the aging process, the following limitations and considerations should be noted. First, we only explored the age-related effects in hemispheric functional interactions using resting-state fMRI data; however, the resting-state functional connectivity is constrained by the brain structural architecture (e.g., white matter streamlines [14, 53]). Thus, it is a promising topic to investigate whether the changes in hemispheric functional patterns with aging are also associated with changes in structural connectivity. Furthermore, no conclusion could be drawn regarding the functional significance of the hemispheric interactions with aging in view of the lack of assessment of cognitive function indicators, such as executive function and fluid intelligence. Researchers are urged to determine the functional role of these hemispheric interactions in the aging process, such as the brain mechanism of cognition decline or adaptation to aging. Third, the symmetrical template used in this study was derived from the AAL atlas that was based on anatomic landmarks and may not fully represent the functional diversity in the brain cortex [54]. Overall, we believe that adopting a hemispheric-level analysis to observe brain network interactions, along with examining multimodal MRI data and behavioral indicators, has the potential to greatly deepen our understanding of brain aging and its relationship to cognition decline in typical aging.

## MATERIALS AND METHODS

### Participants

This sample was acquired from the Southwest University Adult Lifespan Dataset (SALD) exploring the developmental trajectories of brain structural and functional changes in healthy adults [55]. The SALD is available from http://fcon_1000.projects.nitrc.org/indi/retro/sald.html. The dataset contains 494 participants (308 females, aged 19 to 80 years), in which 60 participants were excluded according to a rigorous criterion for framewise displacement (FD > 0.2 mm; [7, 56]). Thus, the final sample was composed of 434 subjects (269 females; mean age = 44.44, SD = 17.28; age range = 19–80). All participants met the MRI-related exclusion criteria and did not have a history of psychiatric disorders, neurological disorders and psychiatric drug use (within the three months before scanning). This project was approved by the Ethics Committee of the Brain Imaging Center Review Board of Southwest University, and written informed consent was obtained for each participant.

### Image acquisition and preprocessing

The MRI data were collected from a 3-T Siemens Magnetom Trio scanner (Siemens Medical, Erlangen, Germany) at the Brain Imaging Research Central in Southwest University. The resting-state functional images were acquired using gradient echo-planar imaging (GRE-EPI) sequences with the following parameters: repetition time (TR)/echo time (TE) = 2000/30 ms, slices = 32, flip angle = 90 degrees, field of view = 220× 220 mm^2^, resolution matrix = 64×64, thickness = 3 mm, interslice gap = 1 mm, and acquisition voxel size = 3.4 × 3.4 × 4 mm^3^. During resting-state scanning, the subjects were instructed to remain awake with eyes closed and rest without thinking of anything in particular. Additionally, high-resolution T1-weighted structural images were obtained using a magnetization-prepared rapid gradient echo (MPRAGE) sequence: TR = 1900 ms, TE = 2.52 ms, inversion time = 900 ms, flip angle = 9°, resolution matrix = 256 × 256, slices = 176, thickness = 1.0 mm, and voxel size = 1 × 1 × 1 mm^3^.

Image preprocessing was performed using the Data Processing and Analysis for Brain Imaging (DPABI, [57]) implemented in the MATLAB 2016a (Math Works, Natick, MA) platform. Preprocessing steps included discarding the first 10 functional images, correcting for slice timing and head motion, co-registering functional data to the Montreal Neurological Institute (MNI) space via T1 image unified segmentation, spatially smoothing with a 4-mm full-width at half-maximum Gaussian kernel, bandpass filtering (0.01–0.1 Hz), and regressing out the confounding signals (white matter and cerebrospinal fluid) and 24 motion parameters [57]. Global signal correction was not employed considering that inter-hemispheric segregation and integration were computed using the functional connectivity between homotopic regions [58].

### Functional network construction and community detection

An absolutely symmetrical template excluding the cerebellum with 512 regions in each hemisphere was applied to calculate intra- and inter-hemispheric functional connectivity [59, 60]. First, region-wise SNR was measured via mean time series divided by the s.d. of the time series, and 25 paired regions were excluded due to inadequate signal (SNR > 2 s.d. above or < 2 s.d. below the group mean). Subsequently, a correlation matrix was calculated by correlating the time series of the remaining ROIs in each hemisphere for each subject. The Louvain method for community detection was adopted using relevant functions from the Brain Connectivity Toolbox [61]. First, the correlation matrix was Fisher z-transformed, resulting in data that were normally distributed, and then empirical thresholding (0.2) was used to remove negative and weak functional connectivity values. Second, the optimal partitioning of all nodes in each hemisphere was detected by using the Louvain community detection algorithm with 150 repeated times in each subject to produce a stable and consensus matrix and avoid a stochastic partition [62]. Finally, the sum of the agreement matrices of the two hemispheres was calculated using a consensus algorithm (100 repetitions) to obtain more accurate partitions [63]. This method resulted in symmetrical networks in the two hemispheres.

### Hemispheric segregation and integration

In this study, we defined a measure of inter-hemispheric segregation as a way of quantifying the differences in within-hemisphere connectivity for a given network in relation to its between-hemisphere connectivity. Specifically, for a given network, within-hemisphere connectivity was estimated by summing the z-values of the correlations between nodes within the network to nodes in the same hemisphere. Conversely, between-hemisphere connectivity was calculated by summing the z-values of the correlations between nodes within the network and nodes located in the contralateral network. The inter-hemispheric segregation was computed with the differences in the within-hemisphere summed magnitudes and the between-hemisphere summed magnitudes as a proportion of the within-hemisphere summed magnitudes, as noted in the following formula:

$$hemispheric\;segregation=\frac{\sum Z_{ll}-\sum Z_{lr}}{\sum Z_{ll}}$$

where $\sum^{\text{​}}Z_{ll}\;$ means the summed Fisher z-transformed correlations between nodes within a given network with all nodes in the left hemisphere, and $\sum^{\text{​}}Z_{lr}$ means the summed Fisher z-transformed correlations between nodes within a given network in the left hemisphere with all nodes in the contralateral network. Higher values for inter-hemispheric segregation indicated that the network was connected to intra-hemispheric nodes to a greater extent, whereas lower values in hemispheric segregation indicated that the network was largely connected to nodes in the contralateral hemisphere. In addition, $\sum^{\text{​}}Z_{lr}$ also indicated the inter-hemispheric interaction for a given network located in the left hemisphere towards the right side.

In addition, intra-hemispheric integration was evaluated by extending the measure of participation coefficient, which reflects the extent to which a node interacts with nodes in other networks. In this study, the intra-hemispheric integration of a network was calculated as the mean participation coefficient [64] across all nodes in this network within each hemisphere. Higher values indicated that the network was more likely to take in other nodes from other networks to reorganize its system, whereas lower values indicated less communication with other networks within the same hemisphere.

### Statistical analysis

First, we examined the Pearson correlation between inter-hemispheric segregation and age after controlling for the participant’s sex and mean FD. Then, we estimated the relationship between age and intra-hemispheric integration after controlling for the participant’s sex and mean FD using Pearson correlation. For all analyses, the Bonferroni correction was used for multiple comparison corrections at α = 0.05. Finally, we explored dynamic brain interactions at the system level across the lifespan by community detection. More specifically, we calculated the differences in the community assignments of nodes across the three age groups (early and middle adult: 19-40 years; late adult: 41-60 years; and old: 61-80 years).

## Supplementary Material

Supplementary Figures

Supplementary Table 1
