# Supplementary material for: Decreased inter-hemispheric interactions but increased intra-hemispheric integration during typical aging
Source: Aging (Albany NY). 2019 Nov 21;11(22):10100–15. doi: 10.18632/aging.102421 (PMC6914428; doi:10.18632/aging.102421)
Supplement: Supplementary Figures [file aging-11-102421-s002..pdf]

SUPPLEMENTARY FIGURES

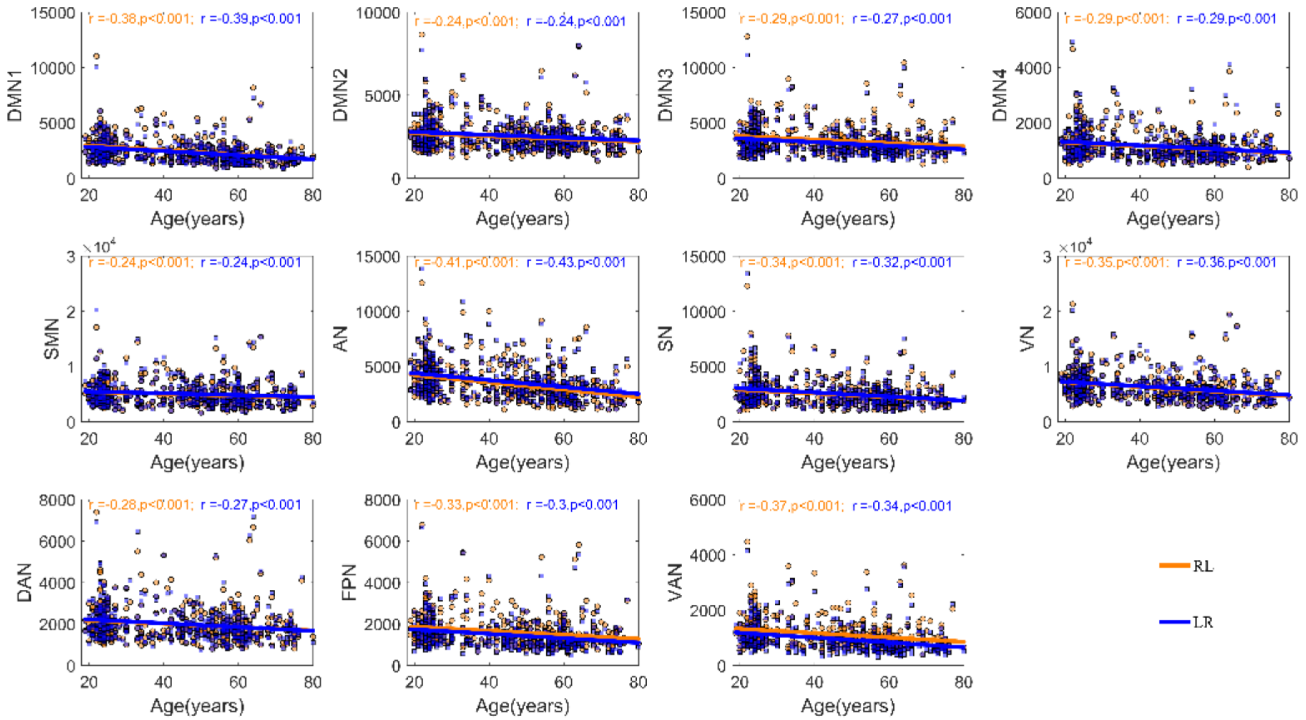

Supplementary Figure 1. The relationship between age and inter-hemispheric functional connectivity for each network after controlling for the participant's sex and mean FD. The blue fitted line indicates a given network in the left hemisphere interacted with all regions within the right hemisphere; the orange fitted line indicates a given network in the right hemisphere interacted with all regions within the left hemisphere.

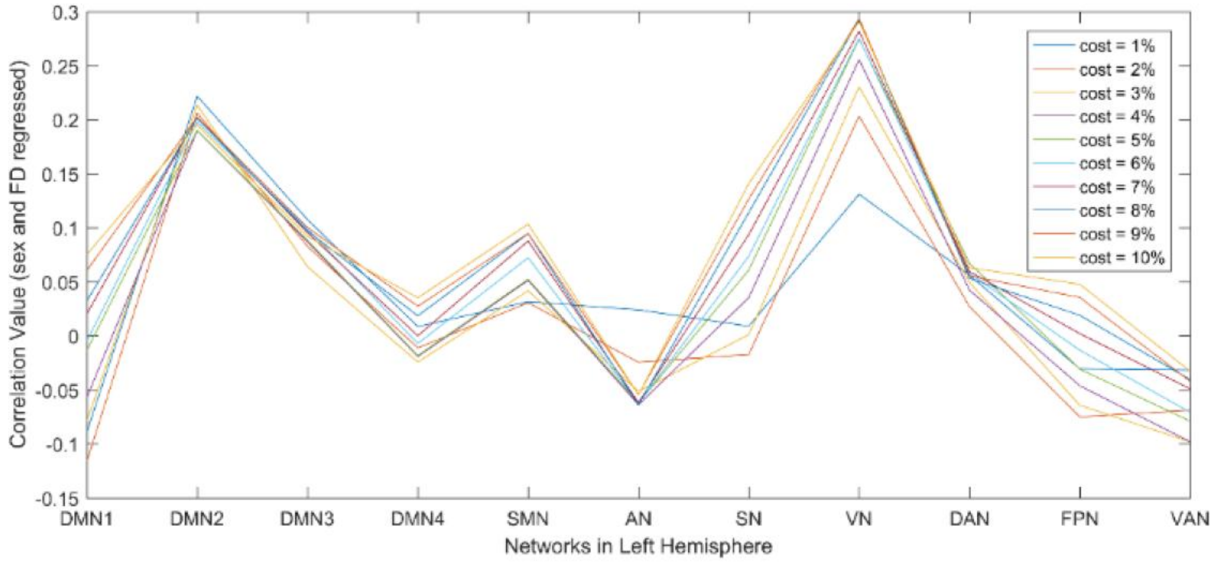

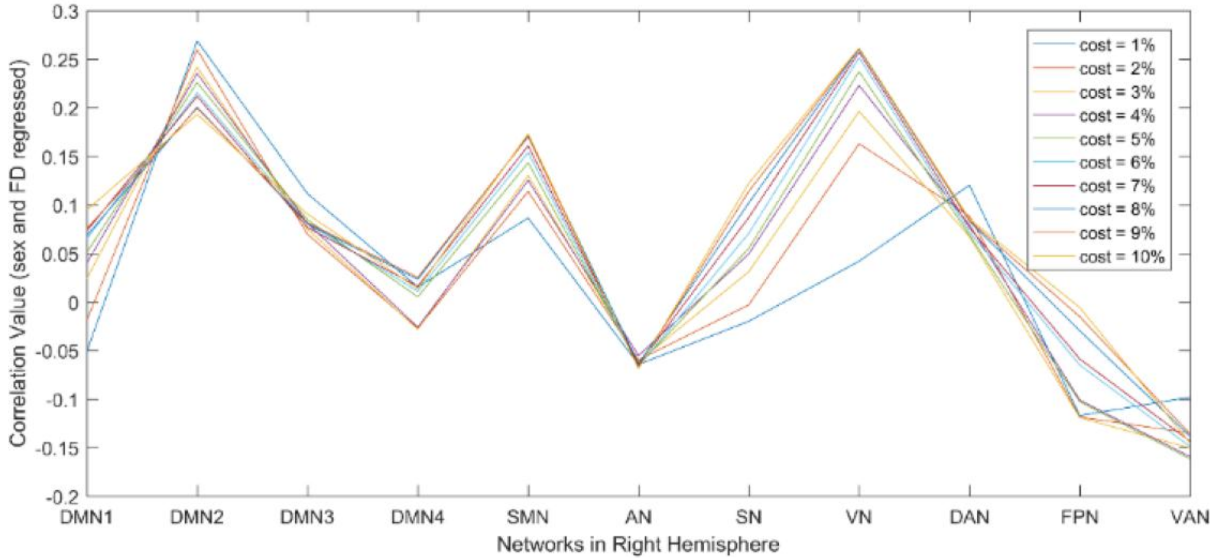

Supplementary Figure 2. The relationship between age and intra-hemispheric integration in the left hemisphere (up) and right hemisphere (bottom) across a range of edge densities (1–10%).
