# Supplementary material for: Decreased inter-hemispheric interactions but increased intra-hemispheric integration during typical aging
Source: Aging (Albany NY). 2019 Nov 21;11(22):10100–15. doi: 10.18632/aging.102421 (PMC6914428; doi:10.18632/aging.102421)
Supplement: Supplementary Table 1 [file aging-11-102421-s001..pdf]

SUPPLEMENTARY TABLE

Supplementary Table 1. Correlation coefficients of age and inter-hemispheric segregation in each hemisphere for 11 identified brain systems after controlling for the participant's sex and mean FD.

Brain system	Left hemisphere		Right hemisphere	
	r	***p***	***r***	***p***
DMN1	0.08	0.102	0.30*	<0.001
DMN2	0.10	0.041	0.15	0.002
DMN3	0.15	0.001	0.15	0.001
DMN4	0.17*	<0.001	0.12	0.01
SMN	0.08	0.107	0.17*	<0.001
AN	0.03	0.501	0.37*	<0.001
SN	0.11	0.017	0.09	0.07
VN	0.12	0.010	0.16	<0.001
DAN	0.19*	<0.001	0.19*	<0.001
FPN	0.30*	<0.001	0.16	<0.001
VAN	0.31*	<0.001	0.15	0.001

Note: * Bonferroni correction ($p<0.01$).
